# LncRNA GAS5 as an Inflammatory Regulator Acting through Pathway in Human Lupus

**DOI:** 10.2174/1381612829666230517102205

**Published:** 2023-06-20

**Authors:** Jianping Xiao, Deguang Wang

**Affiliations:** 1 Department of Nephrology, The Second Affiliated Hospital of Anhui Medical University, Hefei, Anhui, China

**Keywords:** Long noncoding RNA, GAS5, systemic lupus erythematosus, MAPK pathway, cytokines, chemokines, monocytes

## Abstract

**Aim:**

To investigate the contribution of GAS5 in the pathogenesis of SLE.

**Background:**

Systemic Lupus Erythematosus (SLE) is characterized by aberrant activity of the immune system, leading to variable clinical symptoms. The etiology of SLE is multifactor, and growing evidence has shown that long noncoding RNAs (lncRNAs) are related to human SLE. Recently, lncRNA growth arrest-specific transcript 5 (GAS5) has been reported to be associated with SLE. However, the mechanism between GAS5 and SLE is still unknown.

**Objective:**

Find the specific mechanism of action of lncRNA GAS5 in SLE.

**Methods:**

Collecting samples of the SLE patients, Cell culture and treatment, Plasmid construction, and transfection, Quantitative real-time PCR analysis, Enzyme-linked immunosorbent assay (ELISA), Cell viability analysis, Cell apoptosis analysis, Western blot.

**Results:**

In this research, we investigated the contribution of GAS5 in the pathogenesis of SLE. We confirmed that, compared to healthy people, the expression of GAS5 was significantly decreased in peripheral monocytes of SLE patients. Subsequently, we found that GAS5 can inhibit the proliferation and promote the apoptosis of monocytes by over-expressing or knocking down the expression of GAS5. Additionally, the expression of GAS5 was suppressed by LPS. Silencing GAS5 significantly increased the expression of a group of chemokines and cytokines, including IL-1β, IL-6, and THFα, which were induced by LPS. Furthermore, it was identified the involvement of GAS5 in the TLR4-mediated inflammatory process was through affecting the activation of the MAPK signaling pathway.

**Conclusion:**

In general, the decreased GAS5 expression may be a potential contributor to the elevated production of a great number of cytokines and chemokines in SLE patients. And our research suggests that GAS5 contributes a regulatory role in the pathogenesis of SLE, and may provide a potential target for therapeutic intervention.

## 1 INTRODUCTION

Systemic lupus erythematosus (SLE) is a chronic inflammatory disease wherein the immune system attacks healthy cells and tissues throughout the body and is characterized by the production of a large number of autoantibodies, defective elimination of antibodies, circulation and tissue deposition of immune complexes, and activation of the complement system and release of cytokines, ultimately resulting in multiple organ system damage [1-3]. Lupus nephritis (LN) is the most common and serious complication of SLE and has a significant impact on the mortality of SLE patients [4]. Despite extensive efforts to understand the mechanism involved in SLE, the exact pathogenesis of the disease remains unclear.

Multiple studies have indicated that the development of SLE is multifactorial, involving genetic, epigenetic, and environmental factors [3, 5]. An increasing number of studies have recently focused on investigating the role of long noncoding RNAs (lncRNAs) in SLE. Although only 2% of the human genome encodes for proteins, primary transcripts cover 75% of the genome, with processed transcripts covering 62.1% of the genome [6]. This suggests that the functions of lncRNAs are likely underestimated. LncRNAs are defined as transcripts greater than 200 nucleotides in length without an evident protein-coding function. These molecules are associated with diverse biological processes, including epigenetic regulation and gene transcription. The dysregulation of lncRNAs has been associated with various human diseases, including neurological disorders, autoimmune diseases, and cancers [7-11]. However, our understanding of the relationship between lncRNAs and SLE is limited.

The lncRNA Growth arrest-specific transcript 5 (GAS5) has gained much attention in recent research because of its anti-cancer effect. Previous studies have reported that GAS5 can contribute to the resistance function in various types of cancers, including gastric cancer, lung cancer, renal cancer, and hepatocellular carcinoma [12-15]. Moreover, GAS5 has been identified as a potent inhibitor of the glucocorticoid receptor, which plays a key role in regulating the inflammatory process initiated by the immune response [16]. Despite these findings, the role and mechanism of lncRNA GAS5 in SLE remain unclear.

In this study, we observed a significant decrease in the expression level of GAS5 in monocytes from patients with SLE. We further discovered that GAS5 serves as a response gene to LPS and is downregulated by LPS stimulation. Furthermore, overexpression of GAS5 inhibits the proliferation and induces the apoptosis of THP-1 cells. Our results also indicate that GAS5 regulates the expression of inflammatory chemokines and cytokines through the MAPK pathway. In brief, our findings provide insight into the relevance and mechanism of lncRNA GAS5 in the context of SLE.

## 2 MATERIALS AND METHODS

### 2.1 Materials

The THP-1 cells were obtained from the Shanghai Cell Bank of the Chinese Academy of Sciences (Shanghai, China). Trizol was purchased from Invitrogen (Massachusetts, USA). The reverse transcription kit TransScript one-step gDNA Removal and cDNA Synthesis SuperMix were purchased from TransGen Biotech (Beijing, China). SYBR kit was purchased from Bio-Rad (California, USA). PI/Annexin V-FITC apoptosis detection kit was purchased from Becton Dickinson (New Jersey, USA). RIPA lysate (including 1 mmol/L PMSF) was purchased from Nanjing Kaiji Biotechnology Company (Jiangsu, China). The primary antibodies for GAPDH (#5174), JNK (#4672), p-JNK (#4671), p38(#2387), and p-p38 (#2281) were purchased from Cell Signaling Technology (Boston, USA). The primary antibodies for ERK1/2 (ab184699) and p-ERK (ab201015) were purchased from Abcam (MA, USA). Sheep anti-rabbit and sheep anti-mouse secondary antibodies labeled with horseradish peroxidase were purchased from Cell Signaling Technology Company (Boston, USA).

### 2.2 Patients and Healthy Controls

A total of 24 patients with SLE and 25 healthy individuals of similar age were recruited. The patients with SLE were diagnosed according to the 1997 revised American College of Rheumatology (ACR) diagnostic criteria [17]. Disease activity was quantified with the Systemic Lupus Erythematosus Disease Activity Index 2000 (SLEDAI-2K) score. The patients were categorized into active disease (scores > 10) or inactive disease (scores ≤ 10) groups according to the SLEDAI-2K results [18].

This research was approved by the Ethics Committee of Anhui Medical University. All study participants signed the informed consent form.

### 2.3 Cell Culture and Treatment

The HEK293T/THP-1 cells were cultured in Dulbecco’s Modified Eagle Medium (DMEM)-high glucose (Sigma)/RPMI 1640 medium (Gibco, Life technology) supplemented with 10% fetal bovine serum (FBS; Gibco, Life technology), 100 U/mL penicillin, and 100 μg/mL streptomycin at 37°C in 5% CO_2_ atmosphere as described previously [19].

THP-1 cells were cultured in 24-well flat-bottomed plates at a concentration of 5 × 10^5^ cells in 1 mL of complete RPMI 1640 medium and were stimulated with different concentrations of LPS (Sigma) for indicated time points.

### 2.4 Plasmid Construction and Transfection

A GAS5 overexpression plasmid was constructed by inserting a full-length sequence of GAS5 into the pCDH-CMV-MCS-EF1-Puro vector *via* PCR/restriction digest-based cloning. The lentiviral system was generated as follows: oligonucleotides of small interfering RNA for GAS5 (shGAS5) and scrambled control (shSc) were annealed and subcloned into the lentiviral vector pLKO.1. Further, HEK 293T cells were transfected with lentiviral vector and packaging vectors (pVSVG, pREV, and pMDL). The viral supernatants were collected after 48 h of transfection and then used to infect the target cells.

### 2.5 Quantitative Real-time PCR Analysis

The total RNA was extracted from cells using Trizol, according to the protocol provided by the manufacturer. First-strand cDNA was generated using the TransScript One-Step gDNA Removal and cDNA Synthesis SuperMix, according to the manufacturer's instructions, and served as a template. The expression of GAPDH was determined and used as an internal control. Relative expression levels were calculated by the comparative threshold cycle (Ct) method using the formula 2^-ΔΔCt^. More than three independent biological samples were quantified in technical duplicates and expression values were normalized to GAPDH. Nucleotide sequences of the primers used for PCR amplifications were as follows: GAS5 forward sequence: 5’-ATGGTGGAGTCCAACTTGCC-3’, GAS5 reverse sequence: 5’-TCCACACAGTGTAGTCAAGCC-3’; GAPDH forward sequence: 5’- GTCATCCATGACAACTTTGG-3’, GAPDH reverse sequence: 5’- GAGCTTGACAAAGTGGTCGT-3’.

### 2.6 Enzyme-linked Immunosorbent Assay (ELISA)

The levels of various cytokines including interleukin 1-beta (IL-1β), interleukin-6 (IL-6), and tumor necrosis factor-alpha (TNF-α) were measured using corresponding ELISA kits (Genzyme Techne, USA), according to the manufacturer’s protocols.

### 2.7 Cell Viability Analysis

The CCK-8 Assay Kit was used to detect cell viability. In brief, THP-1 cells were plated at a density of 5 × 10^3^ cells/well into 96-well plates in triplicates. Subsequently, 10 μL of CCK-8 solution was added to each well and the plate was incubated for 1-4 h in a cell culture incubator. The absorbance at 450 nm was detected using a plate reader. Cell viabilities were evaluated as relative values compared with the controls. The experiment was repeated three times and the mean and SD values were considered.

### 2.8 Cell Apoptosis Analysis

Cell apoptosis was assessed as described previously by flow cytometry [19]. The THP-1 cells were seeded at a density of 2 × 10^5^ cells/well into 6-well plates. The cells were subsequently treated with various concentrations of LPS for the indicated time points. The cells were then collected *via* centrifugation. After centrifuging at 500 × g for 5 min at room temperature, the supernatant was discarded, and the cells were resuspended with PBS and centrifuged again. Subsequently, the cells were resuspended in 500 μL of binding buffer and 5 μL Annexin V-FITC and 5 μL of PI were added. The cells were incubated for 15 min on a shaker at room temperature in the dark, and the apoptosis of cells in each group was detected by flow cytometry.

### 2.9 Western Blot

Western blot was performed as described previously. The cells were collected and lysed in RIPA cell lysate which contains 1 mM PMSF to extract total protein. The supernatant was obtained by centrifuging at 12000 × g for 5 min at 4°C. The concentration of each tube protein was detected by a BCA protein quantitative kit, and 40 μg protein of each sample containing loading buffer was separated by 10% SDS-PAGE gel electrophoresis, and then transferred to a PVDF membrane (Invitrogen, USA). The membrane was blocked with 5% nonfat milk powder in Tris-buffered saline tween (TBST) at room temperature for 1 h. The membrane was then incubated with the primary antibody on a shaker at 4^o^C overnight. Next, the membrane was washed 3 times in TBST for 7 min each time and then incubated with horseradish peroxidase (HRP) labeled goat anti-rabbit IgG (1:1000 diluted) on a shaker at room for 2 h. After washing, Immunoreactive protein bands were visualized using PierceTM ECL Western Blotting Substrate (Thermo Scientific, Massachusetts, USA) and analyzed by Automatic Gel Imaging System (BIO-RAD, USA).

### 2.10 Statistical Analysis

All statistical analyses were performed using SPSS 20.0. The data are presented as the mean ± SD deviation from more than three individual experiments. Statistical significance was assessed using Student’s t-test or one-way ANOVA. The *P*-value < 0.05 was defined as indicating statistical significance.

## 3 RESULTS

### 3.1 GAS5 Expression is Decreased in SLE Patients

**Fig. (1):**
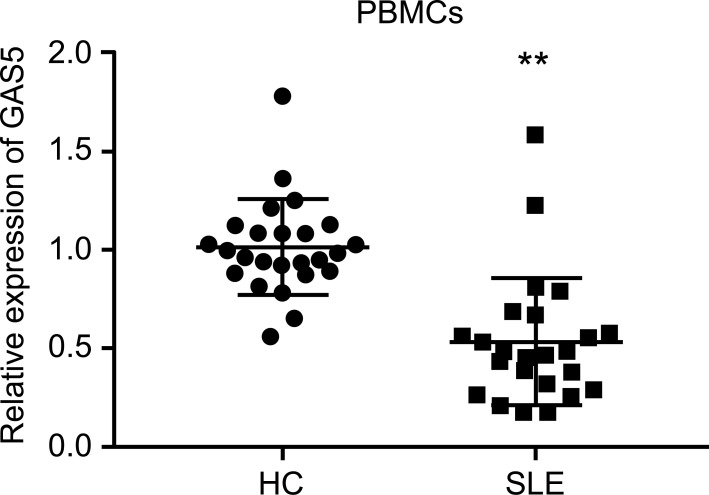
GAS5 expression is decreased in SLE patients. Expression of GAS5 in PBMCs of SLE patients (n = 24) and healthy controls (HC, n = 25) as determined by qRT-PCR analysis. **, *P* < 0.01.

Wu *et al.* found that GAS5 expression decreased significantly in SLE patients compared with that in healthy individuals (*p* < 0.001) using bioinformatics; their study suggested that GAS5 may serve as a potential biomarker for SLE [20]. To verify this conclusion, we collected the plasma samples of 24 SLE patients and 25 healthy individuals from the hospital and performed qRT-PCR to test the GAS5 expression levels. The results showed that the expression of GAS5 in SLE patients was significantly lower than that of GAS5 in healthy individuals. (Fig. **1**) These results suggest that there is a strong correlation between the expression of GAS5 and SLE; however, whether GAS5 is involved in the pathogenesis of SLE is unclear, and further research on the mechanism is needed.

### 3.2 GAS5 Inhibits the Proliferation and Induces the Apoptosis of Monocytes

**Fig. (2):**
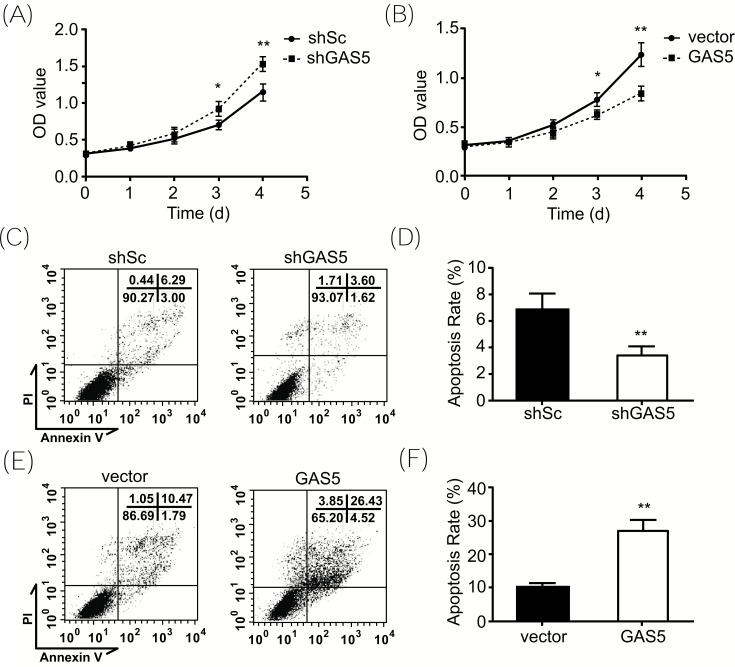
GAS5 inhibits the proliferation and induces the apoptosis of monocytes. (**A** and **B**). Viability of GAS5-knockdown or -overexpression THP-1 cells by CCK-8 assay. (**C**-**F**). Apoptosis of GAS5-knockdown or -overexpression THP-1 cells by flow cytometry. Data are from three independent experiments and expressed as mean ± SD. **, *P* < 0.01.

To further explore the effect of the decreased expression of GAS5 in peripheral monocytes in SLE patients, we constructed GAS5 knock-down THP-1 cell lines using shRNA. THP-1 is a human monocytic cell line and has been extensively used to study innate immune responses. In the process of cell culture, we found that the knock-down of GAS5 in THP-1 cells led to better growth rates. Accordingly, we speculated that GAS5 is involved in regulating the growth, proliferation, and death of THP-1 cells. Therefore, we tested the cell viability of GAS5 knock-down THP-1 cells and control cells through the CCK-8 assay, and the results indicated that knocking down GAS5 makes THP-1 cells have better survival and proliferation characteristics (Fig. **2A**). Similarly, we verified the results by constructing GAS5 overexpression THP-1 cell lines. As expected, the results showed that GAS5 overexpression could inhibit the viability and proliferation of THP-1 cells (Fig. **2B**). Subsequently, apoptosis was evaluated using the PI/Annexin V-FITC apoptosis detection kit combined with flow cytometry. Compared with the control cells, GAS5 overexpression THP-1 cells showed a higher apoptosis ratio (Figs. **2C**-**F**). Therefore, we speculate that GAS5 participates in the immune response by regulating the survival status of peripheral monocytes.

### 3.3 LPS Inhibits GAS5 Expression

**Fig. (3):**
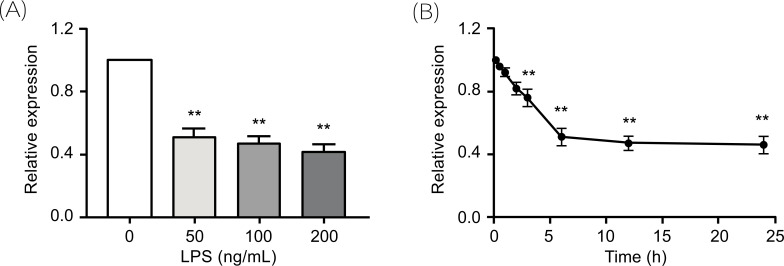
GAS5 expression is suppressed by LPS. (**A**) Analysis of GAS5 expression in THP-1 cells in response to treatment with various concentrations of LPS (0, 50, 100, and 200 ng/mL) for 6 h by qRT-PCR. (**B**) Analysis of GAS5 expression in THP-1 cells following treatment with 50 ng/mL LPS for various durations (0, 0.5, 1, 2, 3, 6, 12, and 24 h) by qPCR. GAS5 expression was analyzed by qRT-PCR, and the bars indicate the mean ± SD from three independent experiments. **, *P* < 0.01.

Emerging evidence reports that the stimulation of TLRs contributes to the initiation and development of lupus disease [21, 22]. Therefore, we wondered whether the activation of TLR signaling in SLE patients is related to decreased expression of GAS5 in monocytes. We stimulated THP-1 cells with LPS, which binds the TLRs in many cell types and promotes the secretion of pro-inflammatory cytokines, and found that the expression of GAS5 was significantly decreased (Figs. **3A** and **B**). These results further indicated that there is a close relationship between the activation of an immune response and the decreased expression of GAS5.

### 3.4 GAS5 Suppresses Inflammatory Cytokine Production Stimulated by LPS

**Fig. (4):**
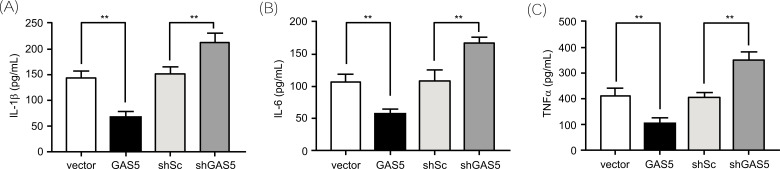
GAS5 suppresses the inflammatory cytokine production stimulated by LPS. (**A**-**C**). The levels of IL-1β, IL-6, and TNFα in cell supernatants as measured by ELISA. Each experiment was repeated three times independently, and the data are expressed as mean ± SD. **, *P* < 0.01.

Considering GAS5 was down-regulated in response to LPS treatment, we were curious about whether GAS5 is involved in the regulation of LPS-induced production of numerous inflammatory cytokines, including IL-1β, IL-6, and TNFα. To reveal the role of GAS5 in the production of inflammatory cytokines induced by LPS, we stimulated the GAS5 knock-down THP-1 cells and the GAS5 overexpression THP-1 cells with LPS and measured the IL-1β, IL-6, and TNFα levels by ELISA. The results showed that silencing GAS5 significantly increased the production of IL-1β, IL-6, and TNFα stimulated by LPS (Figs. **4A**-**C**). However, overexpressing GAS5 obviously decreased the LPS-induced production of the inflammatory cytokines (Figs. **4A**-**C**).

### 3.5 GAS5 Regulates the Activation of the MAPK Pathway

**Fig. (5):**
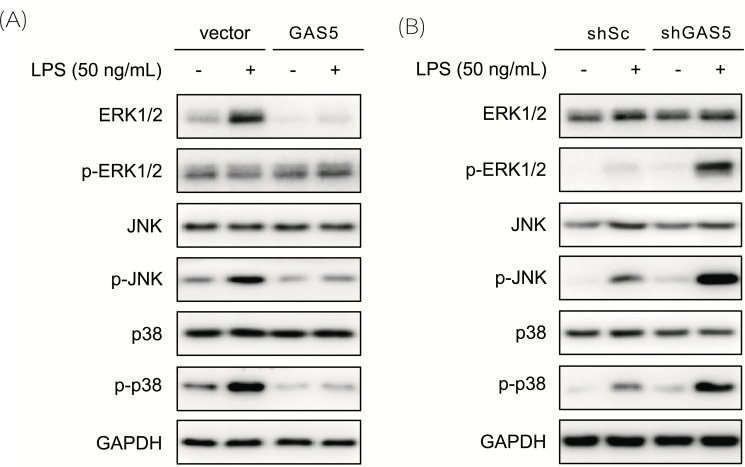
GAS5 affected the MAPK pathway in monocytes. (**A** and **B**). The protein levels of MAPK pathway members as assessed by Western blot assay. GAPDH served as the internal control.

It has been demonstrated that TLR4 stimulation can lead to the activation of the MAPK pathway. In addition, Wu *et al.* found that there is a strong correlation between GAS5 and MAPK pathway [20]; however, whether GAS5 regulates SLE through the MAPK pathway in peripheral monocytes and the exact mechanism between them is unknown. Accordingly, to determine whether GAS5 is involved in the activation of the MAPK pathway, we treated THP-1 cells with LPS and detected the activation of the MAPK pathway. The results showed that MAPK activation in the GAS5 overexpression cells was decreased compared with that in the control cells (Fig. **5A**). As expected, the GAS5 knockdown facilitated the activation of the MAPK pathway induced by LPS (Fig. **5B**).

## 4 DISCUSSION

Innate immune responses are the precursors for all adaptive immune responses, both in normal immunity and autoimmunity, which is the immediate line of defense against both endogenous and exogenous host molecules. Increasing evidence suggests that the innate immune response contributes to the development and regulation of autoimmune diseases [23]. SLE, which is a typical and common autoimmune disease, has a complicated etiology. Abnormal activation of the interferon pathway, stimulation of the TLR pathway, and defective apoptosis are all reported to contribute to the initiation and development of this disease [2].

Monocytes are one of the major components of the innate immune system that are involved in the regulation of an adaptive immune response [24]. Abnormal expression of monocyte surface markers, cytokines, and chemokines in SLE would be a key to its pathogenesis. Monocytes from SLE patients with an active disease display elevated surface levels of intercellular adhesion molecule-1 (ICAM-1, also called CD54) and CD40, which are involved in endothelial transmigration and inflammatory cytokine production [25, 26]. Aberrant activation of the immune system in SLE is augmented by the dysregulated production of inflammatory cytokines and chemokines, and monocytes are a major source of these immunomodulators, which may further promote the activation of autoreactive T and B cells [27]. Monocytes are the primary source of the T helper 2 (Th2) cytokines IL-6 and IL-10 in the peripheral blood, which are elevated in lupus patients [28]. Further, monocytes are a significant source of B-lymphocyte stimulator (BLyS; also known as BAFF), which promotes the survival and proliferation of B lymphocytes. Elevated circulating BLyS/BAFF levels have been detected in nearly 30% of SLE patients, and their levels correlate with the levels of autoantibodies against dsDNA [29, 30].

LncRNAs play multiple biological functions, including chromatin remodeling, gene transcription, RNA splicing, protein translation, and protein transport [31]. LncRNAs serve as a multifunctional regulator of gene expression and function at multiple stages, including at the levels of epigenetic modification, transcription, post- transcription, translation, and post-translation [32]. Recently, an increasing number of studies have revealed that lncRNAs play an important role in regulating the immune response, as well as immune cell development. Increasing evidence suggests that TLRs can recognize various microbial molecules and damage-associated molecular patterns (DAMPs), and ultimately lead to the activation of NFκB and MAPK, which control the expression levels of pro-inflammatory cytokines including IL-1β, IL-6, and TNFα, all of which play crucial roles in immune responses [33]. Guttman *et al.* identified lincRNA Cox2 by stimulating CD11C+ bone marrow-derived dendritic cells with a specific agonist of TLR4 [34]. Further, Zhang *et al.* identified lncRNA NEAT1 as a novel inflammatory regulator through the MAPK pathway in human lupus, which responded to the activation of TLR [35]. Several studies have reported that lncRNAs are involved in the proliferation, development, and apoptosis of immune cells, such as granulocytes, monocytes, and macrophages. Recently, Hao *et al.* demonstrated that the expression of HOTAIR has been significantly up-regulated in AML-*de novo* patients compared with AML-CR patients and normal controls, and HOTAIR knockdown can inhibit the proliferation of AML cells [36]. Hu *et al.* identified LincRNA-EPS, which is required for the terminal differentiation of erythroid cells by inhibiting apoptosis through repressing Pycard [37].

## CONCLUSION

Our findings indicated that the lncRNA GAS5 functions as a negative regulator in TKR4 signaling. As decreased GAS5 expression in monocytes and increased monocytes percentage in PBMCs from SLE patients compared with health controls were observed, we speculated that more monocytes expressed lower GAS5 among PBMCs from SLE patients, producing more inflammatory cytokines and chemokines. Accordingly, GAS5 is negatively correlated to SLE pathogenesis and activity. In addition, GAS5 may contribute to SLE pathogenesis by inhibiting the activation of the MAPK pathway. Furthermore, this research brings us to the knowledge that GAS5 may contribute to a new molecular regulation of autoimmune diseases and may provide insights into the identification of lncRNAs as biomarkers for disease activity and potential therapeutic targets.

## Data Availability

The data and supportive information are available within the article.

## LIST OF ABBREVIATIONS

GAS5: Growth Arrest-specific Transcript 5
LN: Lupus Nephritis
SLE: Systemic Lupus Erythematosus
SLEDAI-2K: Systemic Lupus Erythematosus Disease Activity Index 2000
Th2: T helper 2
